# Functional role of miR-34a in diabetes and frailty

**DOI:** 10.3389/fragi.2022.949924

**Published:** 2022-07-18

**Authors:** Pasquale Mone, Antonio de Donato, Fahimeh Varzideh, Urna Kansakar, Stanislovas S. Jankauskas, Antonella Pansini, Gaetano Santulli

**Affiliations:** ^1^ Division of Cardiology, Department of Medicine, Albert Einstein College of Medicine, New York, NY, United States; ^2^ ASL Avellino, Avellino, Italy; ^3^ BIOGEM, Ariano Irpino, Italy; ^4^ Department of Molecular Pharmacology, Einstein Institute for Aging Research, Einstein-Sinai Diabetes Research Center, Albert Einstein College of Medicine, New York, NY, United States

**Keywords:** miRNA, miR-34, frailty, diabetes, aging

## Abstract

Emerging evidence has shown that microRNAs (miRNAs) play critical role in the pathogenesis of several disorders. In the present minireview, we focus our attention on the functional role of a specific miRNA, namely miR-34a, in the pathophysiology of frailty and diabetes mellitus. Based on the current literature, we speculate that this miRNA may serve as a potential biomarker of frailty in diabetic older adults. Additionally, its actions on oxidative stress might represent a druggable target to obtain new potentials treatments.

## Background

Frailty is a clinical burden which is typical of older adults (Clegg et al., 2013; Hanlon et al., 2018). Frail older adults have a high risk of adverse events such as functional and cognitive impairment, hospitalizations, and death (Puts et al., 2017; Gilbert et al., 2018; Hoogendijk et al., 2019; Pilotto et al., 2020). A prompt diagnosis and a careful management of comorbidities is the first step to avoid adverse outcomes; diabetes is one of these comorbidities and it is very common in frail older adults (Umegaki, 2016; Yarnall et al., 2017; Clegg and Hassan-Smith, 2018; Nagai et al., 2018; Li et al., 2019; Sieber, 2019; Mone et al., 2022a; Mone et al., 2022b; Mone et al., 2022d). Indeed, diabetes leads to a higher risk of cardiovascular complications and functional and physical impairment driving adverse outcomes (Fadini and Avogaro, 2010; Prattichizzo et al., 2018; Yun et al., 2018; Jarvie et al., 2019; Yu et al., 2019; Gulsin et al., 2020).

microRNAs (miRNAs) are small non-coding RNAs that act as post-transcriptional gene regulators (Ambros, 2004; Krol et al., 2010; Santulli, 2015; Ferrante and Conti, 2017; Fridrichova and Zmetakova, 2019; Stavast and Erkeland, 2019; Mone et al., 2021b; Mone et al., Forthcoming 2022c); miRNAs exert their activity in many biological processes and have been proposed as biomarkers and therapeutic strategies (Creemers et al., 2012; Wronska et al., 2015; Barwari et al., 2016; Chen et al., 2018; Wong et al., 2018; Mone et al., 2021b; Fonseca et al., 2021; Mone et al., Forthcoming 2022c). Many miRNAs have been associated to mitochondrial dysfunction, inflammation, and oxidative stress and their concentration may vary in physiological conditions (Zhang et al., 2014; Hathaway et al., 2018; Wei et al., 2018; Rusanova et al., 2019; Song et al., 2019; Gambardella et al., Forthcoming 2022). Interestingly, several investigators evidenced the potential roles of microRNAs (miRNAs) in the pathogenesis of frailty (Rusanova et al., 2019; Carini et al., 2021; Lee et al., 2021; Carini et al., 2022; Dowling et al., 2022).

**FIGURE 1:**
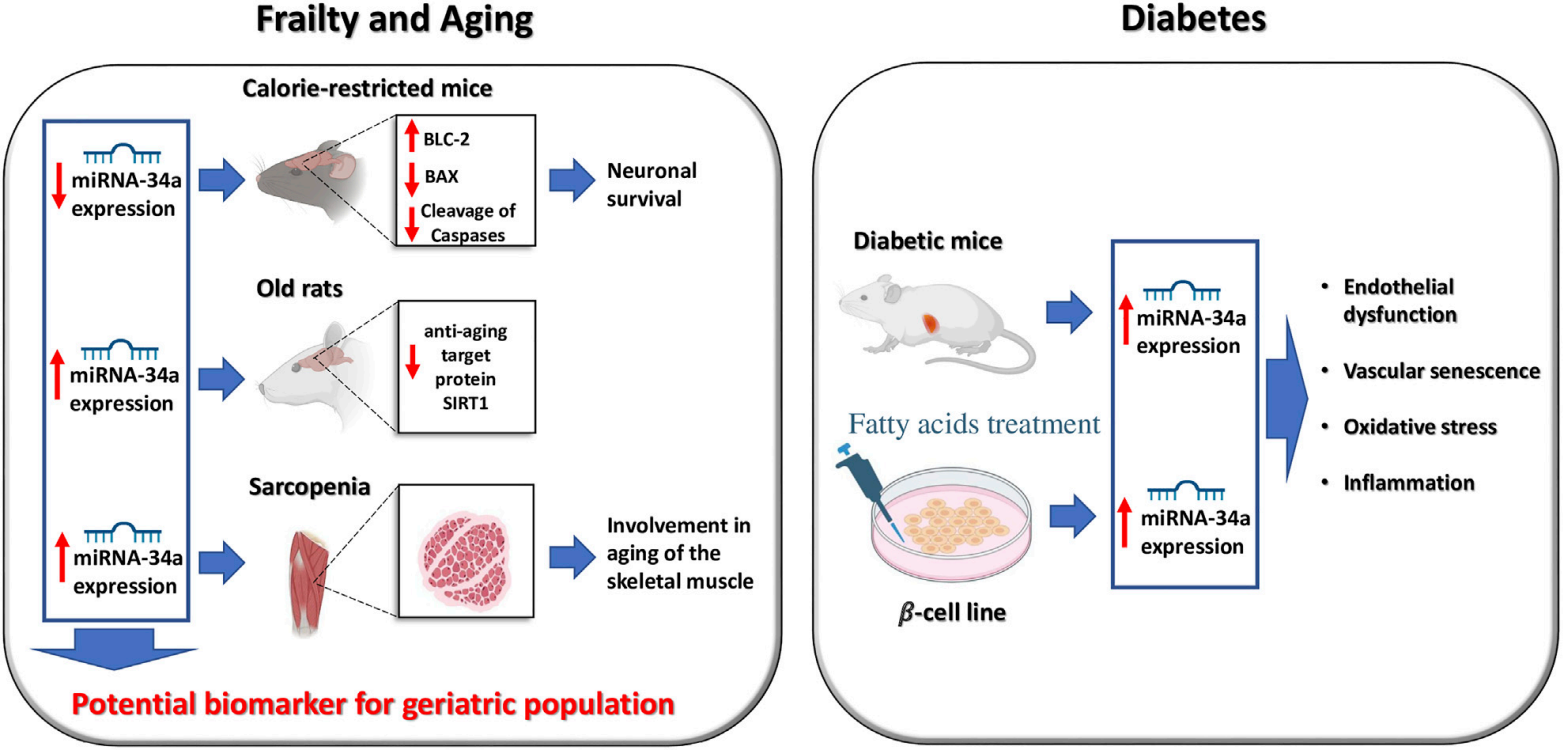
Functional role of miR-34 in frailty, aging, and diabetes.

Specifically, miR-34a has been associated to frailty, aging, and diabetes (Figure 1) (Boon et al., 2013; Chakraborty et al., 2014; Rippo et al., 2014; Badi et al., 2018; Thounaojam and Bartoli, 2019; Kukreti and Amuthavalli, 2020; Ni et al., 2020; Manakanatas et al., 2022) and is generally considered a *bona fide* biomarker of cellular and vascular senescence (Badi et al., 2015; Park et al., 2020; Manakanatas et al., 2022).

### Role of miR-34a in frailty and aging

The pathophysiology of frailty includes chronic inflammation, which is prevailing in aging (“inflammaging”), oxidative stress with or without mitochondrial dysfunction, insulin resistance, loss of anabolic hormones, and reduced tolerance to physical exercise with a reduction in muscle strength (Bandeen-Roche et al., 2015; Cruz-Jentoft and Sayer, 2019; Rusanova et al., 2019).

Frailty onset is due to the failure of multiple organs and/or systems and many pathologic conditions have been associated with frailty (Walston et al., 2008; Afilalo et al., 2014; Mone and Pansini, 2020; Mone et al., 2021a; Waite et al., 2021; Mone et al., 2022e). In 2001, Fried et al. (2001) developed the five criteria now routinely used to diagnose frailty. Equally important, the frailty index is another tool to diagnose and manage frailty (Rockwood et al., 2005; Searle et al., 2008).

In 2011, Khanna et al. (2011) observed an age-dependent decreased expression of miR-34a in the brain of calorie-restricted mice, mirrored by an increase in Bcl-2 expression, and a reduced expression of pro-apoptosis genes such as Bax. The authors concluded that this miRNA was involved in the neuronal survival in long-lived calorie-restricted fed mice.

A subsequent investigation by Zheng and collaborators evidenced the involvement of miR-34a in cellular senescence via MAPK: the authors detected its overexpression in sarcopenia, suggesting a role of this miRNA in the aging process of the skeletal muscle (Zheng et al., 2018). Similarly, miR-34a expression was significantly up-regulated in the hearts of aged mice lacking Calstabin 2, the stabilizing protein of the cardiac isoform of Ryanodine Receptor (Yuan et al., 2014). Another investigation revealed that an increased expression of miR-34a in older rats correlates with a concomitant decrease in the brain of the anti-aging target protein SIRT1 (Hu et al., 2017).

Notably, a clinical paper indicated miR-34a as a biomarker of aging/frailty in oncogeriatric populations (Dalmasso et al., 2018). In line with these observations, a very recent paper evidenced that miR-34 regulates protein translation and protein turnover in the aging brain of *Drosophila* (Srinivasan et al., 2022).

### Role of miR-34a in diabetes

Insulin resistance is one of the most important features of Type 2 Diabetes mellitus (T2DM) (Feve and Bastard, 2009; Taylor, 2013; Mastrototaro and Roden, 2021). Of note, miR-34a supports pancreatic development and has been associated to insulin resistance and to the onset of T2DM (Wei et al., 2013; Chakraborty et al., 2014). Intriguingly, previous investigations had highlighted that the expression of miR-34a is increased in islets of diabetic mice (Rottiers and Naar, 2012). The prolonged exposure of saturated fatty acids to MIN6 β-cells and pancreatic islets increased the expression of miR-34a (Lovis et al., 2008). Furthermore, miR-34a leads to endothelial dysfunction and vascular senescence in diabetes (Li et al., 2016; Carracedo et al., 2019; Thounaojam and Bartoli, 2019), increasing the overall risk of oxidative stress and inflammation with or without diabetes (Li et al., 2016; Cheleschi et al., 2019; Xiong et al., 2019; Zimta et al., 2019; Li et al., 2021; Mahjabeen et al., 2021; Zhu et al., 2021).

## Conclusion

Herein, we summarized the investigations linking miR-34a and frailty. Furthermore, miR-34 may be linked to diabetes and endothelial dysfunction. Based on the provided evidence, we speculate that this miRNA may serve as a potential biomarker of frailty in diabetic older adults. Additionally, its actions on oxidative stress might represents a druggable target in order to develop new potentials therapeutic options.
